# Russian Cure for Drunkenness

**Published:** 1876-04

**Authors:** 


Russian Cure for Drunkenness.—H. Haurowiz says that for some time past *Herba serpylli* (wild thyme) has been used with great success to effect a permanent cure of drunkenness; in case of a relapse (only after years), a short treatment will effect a cure again. The treatment consists in making an infusion of wild thyme (an ounce and a half to a pint and a half), and give the patient a teacupful every half hour. The next day it is given every two hours, and then four to six times a day until the cure is complete, which generally takes from two to three weeks. The effects are in the following order: vomiting, diarrhœa, increased urine, strong transpiration; then generally increased appetite and craving for acidulous beverages. Diet: easily digested food and lemonade or other acidulous liquids.—*Amer. Journ. of Pharm.*

